# Epigenetic Mechanisms in Early Life Viral Respiratory Infections

**DOI:** 10.3390/v18030345

**Published:** 2026-03-12

**Authors:** Juliana Poppe, Katarzyna Placek, Ana Paula Duarte de Souza

**Affiliations:** 1 Laboratory of Clinical and Experimental Immunology, Healthy and Life Science School, Pontifical Catholic University of Rio Grande do Sul (PUCRS), Porto Alegre 90619-900, RS, Brazil; juliana.poppe@edu.pucrs.br; 2 Department of Immunology and Metabolism, Life and Medical Sciences (LIMES) Institute, University of Bonn, 53115 Bonn, Germany; kplacek@uni-bonn.de; 3 Institute for Research on Infectious and Chronic Diseases of the Mucosae and Skin, Belo Horizonte 31270-901, MG, Brazil; 4 Infant Center, School of Medicine, Pontifical Catholic University of Rio Grande do Sul (PUCRS), Porto Alegre 90619-900, RS, Brazil

**Keywords:** early life, epigenetic, respiratory virus

## Abstract

Early-life respiratory viral infections represent a major global health burden and are key determinants of long-term susceptibility to chronic respiratory diseases. In neonates the immaturity of the immune system contributes to the high incidence and severity of these infections. Because humans are born with a mainly naive adaptive immune system, the host protection in early life greatly relies on the innate immune cells. Interestingly, innate immune cells have been recently shown to develop traits of immune memory. Both adaptive and innate immune memory formation are, among others, mediated by epigenetic mechanisms such as DNA methylation, histone modifications, and non-coding RNAs. This review comprehensively analyzes evidence of the changes in epigenetic modifications before and after respiratory infection in childhood. Understanding how epigenetic programming modulates immune cells in early life may open new avenues for preventive interventions to respiratory viral infection, enhancing antiviral defense in infancy and reducing the long-term consequences of respiratory infections.

## 1. Introduction

During the early life, particularly at the first year, children exhibit heightened vulnerability to respiratory infections. This increased susceptibility reflects the functional immaturity of both the innate and adaptive immune systems, which are still undergoing critical developmental processes [1,2]. During this period, there is a lower capacity for activation of antigen-presenting cells, a reduction in the production of type I and III interferons, and a weakened inflammatory response, resulting in less effective control of viral replication [3]. Moreover, there are many regulatory mechanisms and epithelial barriers undergoing maturation, making infants particularly dependent on maternally derived antibodies transferred via the placenta and breast milk [4]. Accordingly, breastfeeding has been consistently associated with enhanced mucosal immunity and reduced incidence of acute respiratory infections, partly due to the transfer of maternal antibodies and bioactive immunomodulatory factors.

The most common viruses affecting this population include respiratory syncytial virus (RSV), human rhinovirus (HRV), adenovirus, human coronavirus, and influenza A and B viruses, followed by parainfluenza virus, metapneumovirus, bocavirus, and cytomegalovirus [5]. Among the respiratory viruses of greatest epidemiological relevance, RSV stands out as the leading etiological agent of lower respiratory tract infections in young children. In 2019, RSV was estimated to cause approximately 33 million cases of acute lower respiratory tract infection in children under five years of age. More recent studies identify RSV as one of the leading causes of hospitalization in this age group, being responsible for more than 100,000 deaths per year worldwide—a number that highlights the significant impact of this virus on child health and global healthcare systems [6].

HRVs rank as the second most frequent group of pathogens in pediatric respiratory diseases and are associated with prolonged hospitalizations in this population. HRVs are the main agents responsible for the common cold and have been increasingly detected in infections of both the upper and lower respiratory tracts [7]. Moreover, HRV infection is linked to the onset and worsening of asthma in children [8,9,10]. The influenza virus is responsible for an acute infectious viral respiratory disease that affects a large portion of individuals. It is periodically responsible for annual influenza epidemics and severe cases in children [11]. Other viruses—such as metapneumovirus, parainfluenza, adenovirus, bocavirus, human coronavirus, and cytomegalovirus—also contribute to the high number of respiratory infections in children [5,12]. Despite the typically rapid recovery without the need for medical intervention, newborns are more likely to develop severe complications, require hospitalization, and may even die [12].

Early-life interventions are available to reduce the burden of respiratory infections in infants and young children. Immunoprophylaxis using monoclonal antibodies, such as palivizumab or the long-acting antibody nirsevimab, has been shown to effectively prevent severe RSV disease in high-risk and healthy infants [13]. Routine childhood vaccinations, including those against influenza, pertussis, pneumococcus, and *Haemophilus influenzae* type b, also play a central role in mitigating both viral and secondary bacterial respiratory infections. Maternal vaccination enhances the transplacental transfer of virus-specific IgG antibodies, providing passive protection to the infant during the first months of life. Phase 3 clinical trials have demonstrated that maternal immunization with the bivalent respiratory syncytial virus (RSV) vaccine administered between 24 and 36 weeks of gestation significantly reduces the incidence of severe lower respiratory tract disease in infants during early life [14,15,16]. Additional public health measures, including reducing exposure to tobacco smoke, improving indoor air quality, and promoting hand hygiene and daycare infection-control practices, further contribute to lowering infection risk during this vulnerable period. Collectively, these interventions form a multifaceted preventive framework aimed at protecting infants while their immune systems continue to mature.

Despite advances in treatment and prevention, the mortality rate among children under five years of age remains high. This highlights the importance of understanding not only the epidemiology and immunology of these diseases but also the potential long-term effects they may have on children’s respiratory health [17]. On the one hand, the neonatal period in life can lead to epigenetic changes in the immune system with the potential to prevent respiratory viral infection later in life; on the other hand, recent studies have suggested that early-life respiratory infections may trigger epigenetic modifications that modulate immune responses throughout life—a topic that underpins the present review.

## 2. Methods

A semi-structured search of the relevant literature was conducted using the databases of PubMed in October of 2025, focusing on peer-reviewed articles. Representative search strings included: (“breastfeeding” OR “breast feeding”) AND epigenetic* AND (“respiratory infection*” OR RSV OR rhinovirus); (microbiota OR microbiome) AND epigenetic* AND (“respiratory infection*” OR RSV OR rhinovirus); and (vaccine* OR vaccination) AND epigenetic* AND (“respiratory infection*” OR RSV OR rhinovirus). Since this is a narrative review, variations in these expressions were applied to capture relevant literature. This search strategy ensured conceptual specificity, relying primarily on the term “epigenetic” which may have limited the retrieval of studies investigating specific molecular mechanisms—such as DNA methylation, histone modifications, chromatin remodeling, or microRNA regulation—that are not always indexed under epigenetic terminology.

## 3. Epigenetic Mechanisms and Trained-Immunity

### 3.1. Epigenetic Regulatory Mechanism

The term “epigenetic” was proposed by Conrad Hal Waddington in 1942, which suggested that the process of cellular differentiation may be regulated by the epigenetic landscape and not by genetic inheritance [18]. Cells employ epigenetic mechanisms to regulate gene expression without altering the DNA sequence. The principal components of this regulatory machinery include DNA methylation, histone modifications, noncoding RNAs.

DNA methylation, one of the most studied epigenetic mechanisms, was first described by Halliday and Pugh in 1975 and has been involved in different biological processes, such as genomic imprinting, genome stability, and transposon silencing [19,20]. DNA methylation consists of the enzymatic process through which methyltransferases transfer a methyl group (–CH_3_) from S-adenosyl-methionine to the fifth carbon of cytosine residues within CpG dinucleotides. During DNA replication methyl groups are established de novo, maintained, or are subsequently removed. The equilibrium between DNA methyltransferases and demethylating enzymes ensures the stability of the methylation pattern [21]. There are five DNMTs (DNMT1, DNMT2, DNMT3A, DNMT3B, and DNMT3L) in mammals, responsible for the maintenance of already existing DNA methylation patterns or establishing a new DNA methylation pattern known as de novo DNMTs. DNA demethylation is performed by the ten-eleven translocation (TET) family of enzymes [22].

The second most studied epigenetic marks are histone modifications. Histones are nuclear proteins responsible for packaging and organizing DNA into chromatin. DNA is wrapped on a complex of 4 histones (H2A, H2B, H3, and H4) together called a nucleosome that serves as the fundamental unit of chromatin structure. In addition to their architectural function, histones undergo numerous post-translational modifications, which regulate chromatin accessibility, recruitment of other proteins and RNA molecules, and gene expression, configuring an important epigenetic mechanism. The most well-studied histone modifications are acetylation and methylation, although there are many others described [23].

Histone acetylation is primarily regulated by histone acetyltransferases (HATs) and histone deacetylases (HDACs) and predominantly occurs on lysine residues located in the N-terminal tails of histones H3 and H4. This reversible modification is one of the most extensively characterized histone marks. HATs catalyze the transfer of an acetyl group to specific lysine residues, thereby promoting histone acetylation and increasing chromatin accessibility. Conversely, HDACs catalyze removal of acetyl groups, leading to chromatin compaction and transcriptional repression [24]. Histone methylation is catalyzed by methyltransferases and removed by demethylases. Depending on the location of the lysine residue modified, histone methylation can have a distinct effect on gene expression. For example, histone 3 (H3) lysine (K) 4 methylation is associated with gene activation while H3K27 methylation suppresses gene expression [25].

A more recently described epigenetic mechanism involves non-coding (nc) RNAs. Advances in sequencing technologies have led to the identification of a vast number of ncRNAs. Based on their structure, ncRNAs are classified into small ncRNAs (sncRNAs; 18–200 nucleotides), long ncRNAs (lncRNAs; >200 nucleotides), and circular RNAs (circRNAs). sncRNAs can be further subdivided into microRNAs (miRNAs), small nuclear RNAs (snRNAs), and PIWI-interacting RNAs (piRNAs). ncRNAs participate in a wide range of biological processes, such as epigenetic regulation of gene expression in different contexts including during immune response [26].

Collectively, these epigenetic mechanisms provide the molecular basis through which environmental and microbial signals induce durable functional changes in immune cells, enabling the sustained modulation of innate and adaptive immune responses over time.

### 3.2. Epigenetic Regulation of the Adaptive Immune Memory Responses

The immune system is conventionally classified into two main components: innate and adaptive immunity. The innate immune system provides the first line of defense, integrating genetically encoded pattern-recognition receptors that detect pathogen-associated molecular patterns and damage-associated molecular patterns, rapidly triggering non-specific and immediate effector programs. These programs include the activation of interferon signaling pathways, inflammasome assembly, and the recruitment of effector myeloid cells. In contrast, the adaptive immune system generates highly specific responses through somatically recombined antigen receptors expressed by B and T lymphocytes. This component is not only crucial for antigen-specific recognition but is also responsible for long-lasting immunological memory, promoting the differentiation of memory B cells, long-lived plasma cells, and memory T-cell subsets, thereby ensuring faster and more efficient responses upon re-exposure to the same pathogen [27,28].

The acquisition of adaptive immune memory is, among others, regulated by epigenetic processes. It is well established that changes in DNA methylation pattern accompany the transition from naïve B and T lymphocytes to memory subsets [29,30,31,32,33]. In general, it has been observed that many regulatory elements of genes involved in differentiation and effector function of lymphocytes while enriched in DNA methylation in naïve cells, lose the DNA methylation status in memory lymphocytes enabling a quick reactivation of the genes upon antigenic reencounter. Similarly, histone modification pattern changes with the acquisition of memory phenotype by B cells [34,35] and T cells [31,36,37,38,39,40,41,42]. Gene loci related to lymphocyte effector function are in general enriched in activating histone marks: H3K4me1, H3K4me3 and H3K27ac in memory cells compared to naïve cells. Concomitantly, genes that are downregulated in effector lymphocytes are deprived of histone activating marks in memory cells but gain the suppressive histone modification H3K27me3. Furthermore, several ncRNAs and noncoding RNA circuits have been linked to memory B cell [34,43] and T cell formation [44,45,46,47,48,49,50,51,52,53] by directly affecting effector molecule production or by interfering with metabolic and signaling pathways. Altogether a large body of evidence demonstrated the importance of epigenetic mechanisms in the acquisition of memory phenotype by adaptive immune cells and their role in efficient recall immune responses.

### 3.3. Epigenetic Rewiring in Trained Immunity

Within the epigenetic context, recent studies have demonstrated that innate immune cells can undergo functional reprogramming following exposure to infectious stimuli, resulting in enhanced responsiveness upon subsequent encounters. This phenomenon, termed “trained immunity”, confers memory-like properties to innate immune cells through epigenetic remodeling, metabolic reprogramming, and transcriptional alterations [27]. The term “trained immunity” was first mentioned in 2011 and describes how innate immune cells, including monocytes, macrophages, and NK cells, can generate a more robust response to secondary stimulation through epigenetic and metabolic reprogramming. Importantly, secondary immune responses of innate immune cells in contrast to adaptive immune cells are not antigen-specific implying enhanced immune response to heterologous stimuli. This enhanced responsiveness is associated with several epigenetic mechanisms, such as histone modifications, DNA methylation, and changes in chromatin accessibility, as described above [27,46,54,55,56].

Epigenetic alterations associated with trained immunity include marks such as H3K4 and H3K27 methylation, as well as DNA methylation of gene loci that are crucial for immune responses [57]. These modifications are fundamental for activating host defense mechanisms and can be maintained long after the initial exposure, rendering responses to subsequent infections more efficient. DNA methylation at gene loci critical for antiviral responses, including those related to type I interferon production, has also been implicated in the generation of trained immunity [21]. Furthermore, whole genome mapping of changes in chromatin accessibility in innate immune cells following multiple pathogen exposures, suggested that increased chromatin accessibility may facilitate rapid and effective immune responses upon re-exposure to the same pathogen [58,59].

Alterations in cellular metabolism are also fundamental to trained immunity. For instance, metabolic reprogramming toward aerobic glycolysis, driven by metabolites, enhances the activity of epigenetic enzymes that regulate histone modifications [28,46,60,61]. These metabolites can act as epigenetic cofactors, promoting histone modifications and altering gene expression to improve immune system responses [60]. For example, fumarate accumulation can inhibit histone demethylases that depend on α-ketoglutarate, maintaining activating histone marks such as H3K4me3 at inflammatory gene loci [28,46]. Increased availability of acetyl-CoA supports histone acetyltransferase activity, enhancing chromatin accessibility, whereas α-ketoglutarate serves as a cofactor for DNA and histone demethylases, contributing to the regulation of immune gene expression [61,62]. Furthermore, increased lactate production during trained immunity induction has been linked to histone lactylation and transcription at gene loci associated with trained immunity phenotype [63].

Although trained immunity can enhance protection against infections, in certain contexts it may also lead to an amplification of inflammatory responses, contributing to autoimmune and inflammatory diseases. The timing and nature of the immunological stimulus are critical determinants of whether trained immunity will be protective or pathogenic [64].

Early-life viral respiratory infections are strongly associated with the development of asthma, a condition increasingly recognized as being influenced by epigenetic mechanisms. Viral exposure during critical developmental windows can induce epigenetic remodeling in immune and airway cells, altering gene expression programs related to inflammation, type 2 immune polarization, and airway hyperresponsiveness [65,66,67,68]. Changes in histone modifications, DNA methylation, and chromatin accessibility have been linked to sustained dysregulation of immune and epithelial responses, contributing to asthma susceptibility. Evidence further suggests that these epigenetic alterations may persist over time, helping to explain why pediatric asthma can extend into adulthood and why some late-onset phenotypes may originate from early-life exposures. In this context, epigenetic programming initiated by early inflammatory events may shape respiratory health throughout life [69,70].

## 4. Epigenetic Mechanisms and Respiratory Infection

**Figure 1:**
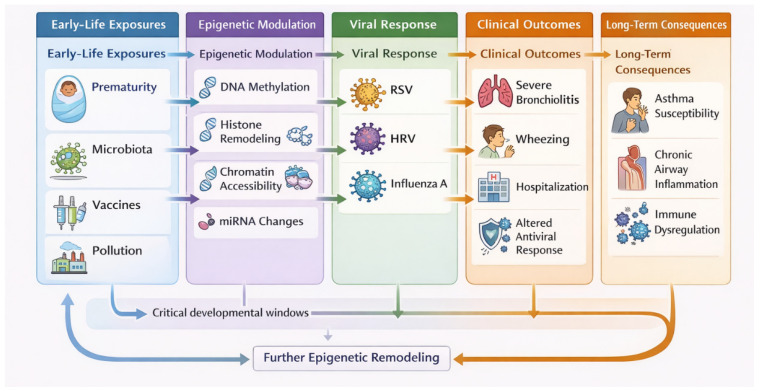
Schematic representation of the influence of early immunomodulatory exposures and epigenetic remodeling on the response to respiratory infections across the lifespan. Exposures during early developmental periods, particularly at birth and in infancy, can induce epigenetic modifications that shape the immune system. These alterations influence the host response to subsequent respiratory infections, affecting disease outcomes in childhood and contributing to long-term consequences throughout life. Also, early-life respiratory infections may trigger epigenetic modifications with lasting effects throughout childhood. Figure was created by the authors using ManusAI v1.6.

Early-life exposures can induce lasting epigenetic modifications in immune cells that influence susceptibility, severity, and resolution of respiratory infections by modulating immune responses. Conversely, respiratory viral infections themselves can imprint long-term epigenetic and transcriptional changes on airway and immune cells, potentially predisposing individuals to chronic respiratory diseases later in life (Figure 1).

### 4.1. Early-Life Immunomodulating Exposures That Alter Epigenetic Patterns Influence the Severity of Respiratory Viral Infection

The pulmonary innate immune system develops rapidly in early life, yet the degree of immune maturation and immune-shaping exposures prior to a child’s first respiratory viral infection critically influence the outcome of severe disease and the risk of long-term airway morbidity. Examples of these immune-shaping exposures are prematurity, microbiota composition, breastfeeding, environmental factors, and vaccines that may be associated with epigenetic modulation. The increased susceptibility for respiratory infection or better resolution of the infection might be associated with prior epigenetic mechanisms in respiratory tract cells.

For example, it has been described that priming human bronchial epithelial cells with IFN-gamma, a key cytokine produced mainly by T cells and NK cells that is essential for antiviral defense, reduced the RSV viral load. Exposure to IFN-gamma on the epithelial cells leaves an epigenetic mark on the chromatin that enhances airway cells’ ability to resist respiratory viral infection via epigenetic upregulation of RIG-I [71].

Prematurity, and the consequent exposure to neonatal intensive care, is known to trigger inflammatory processes and impose substantial physiological stress during a critical developmental window. Cord blood samples from preterm and at-term babies showed differences in DNA methylation pattern in immune-related genes between the two groups suggesting differential epigenetic regulation of inflammatory responses [72]. However, it has been demonstrated that the exposure of preterm born children to intensive care and infections does not accelerate age-related epigenetic changes at DNA methylation level [73]. In contrast, interventions to prevent respiratory infections can lead to modification in DNA methylation later in life. Preterm infants who received the RSV immunoprophylaxis, with a monoclonal antibody against protein F (palivizumab), presented long-lasting epigenetic changes detectable in nasal cells at age six. In particular, children who received palivizumab showed distinct global DNA methylation patterns, including shifts in pathways related to sensory perception and T-cell differentiation, likely reflecting altered cell-type composition [74].

In the first months of life, the microbiota plays an essential role in shaping an efficient and balanced immune system capable of responding appropriately to infections [75]. The development of the microbiota is strongly influenced by environmental factors, such as diet, medication use, living conditions, diseases, and gestational events, as well as the host’s own genetic characteristics. These elements, together, impact microbial composition and, consequently, influence immunological maturation throughout life [76]. This symbiotic relationship with non-pathogenic microorganisms is essential for guiding and reprogramming basic immune functions.

It is not surprising that imbalances in the microbiota during early life have already been associated with an increased risk of allergic diseases, highlighting that microbial interventions may reduce susceptibility to respiratory viral infections [77,78]. Recent studies have shown that alterations in the nasal and intestinal microbiota of infants are associated with a higher risk of respiratory viral infections, including respiratory syncytial virus (RSV), influenza, and rhinovirus. For instance, infant cohorts have demonstrated that reduced diversity of the nasal microbiota increases the likelihood of recurrent respiratory symptoms, while changes in the intestinal microbiota are linked to greater severity of RSV infections [16,78,79,80]. Furthermore, reviews indicate that the microbiota acts as an epigenetic mediator, modulating antiviral immune responses via the gut-lung axis, influencing DNA methylation and chromatin states, which directly impacts host immunity [16]. Thus, microbiota diversity and balance during infancy play a central role in resistance to respiratory infections and in shaping balanced immune responses, highlighting the potential of early-life microbial interventions to prevent viral respiratory diseases.

Early-life antibiotic-induced dysbiosis in mice reduces iodine levels, impairing influenza-specific CD8+ T cell immunity by disrupting NFIL3 (nuclear factor, interleukin-3 regulated)-dependent epigenetic control of T cell factor 1 (TCF-1) expression [81]. Also, in mice, it was suggested that stress increases the susceptibility to respiratory infections (influenza and SARS-CoV-2), in part by disrupting the gut microbiome, particularly reducing Lactobacillaceae abundance and lowering microbial-derived GABA (γ-aminobutyric acid). This effect is mediated by Tet-2 (ten-eleven translocation methylcytosine dioxygenase 2), enabling DNA hydroxymethylation and activating a gene program controlled by peroxisome proliferator-activated receptor gamma that supports the immunoregulatory functions of alveolar macrophages. Restoring GABA-producing bacteria reverses these stress-induced effects and mitigates viral pneumonia severity [82]. Other studies in mice also highlighted the role of gut microbiota and epitranscriptomic regulation on attenuation of influenza-induced pulmonary inflammation suppressing NLRP3 activation, particularly on METTL3-mediated m6A modification of NLRP3 [83].

Environmental factors can contribute to early life susceptibility to respiratory viral infections [84]. Environmental exposures, including daycare attendance, presence of siblings, indoor dampness, environmental tobacco smoke, pet exposure, and air-pollution sources such as traffic and solid-fuel combustion, have all been associated with altered risk of respiratory infections in infancy and childhood. Several of these exposures have also been linked to distinct epigenetic signatures in immune-related pathways. Phthalates, substances added to plastics to increase their flexibility, transparency, and durability, represent a class of environmental chemicals that may influence susceptibility to upper respiratory infections in children [85]. A study demonstrated that higher prenatal exposure to phthalates may be associated with increased susceptibility to early childhood respiratory infections, especially in boys, and epigenetic age acceleration serves as a potential biological mechanism [86].

A pediatric cohort study has shown that children predisposed to recurrent respiratory infections in their first 5 years of life have increased DNA methylation levels at genes associated with innate immune response prior to infections [87]. This suggests a potential role of epigenetics in shaping the susceptibility to respiratory infections. Furthermore, the children that presented this endotype signature of differentially methylated regions were also those who were low vaccine responders measured by antibodies specific against six different vaccines [88].

Bacillus Calmette–Guérin (BCG), a live-attenuated *Mycobacterium bovis* vaccine primarily used for tuberculosis prevention is one of the earliest after-birth given vaccines in many countries. BCG vaccination has been shown to reprogram innate immune cells through epigenetic mechanisms, thereby enhancing innate immune memory and promoting more robust responses to subsequent infections [89]. BCG might have important implications for non-specific prevention of respiratory viral infections in vaccinated children [90].

Similarly, SARS-CoV-2 mRNA vaccines induce lasting epigenetic and transcriptomic reprogramming in monocyte-derived macrophages [91]. Also, miRNA epigenetic modifications significantly influence the development and strength of influenza vaccine-induced immunity [92]. DNA methylation sites induced by Influenza vaccine are described as signatures to predict protection against the virus [93]. Further studies are needed to associate those epigenetic modifications with the protection against respiratory infections in children.

### 4.2. Respiratory Viral Infections in Early Life Leave Lasting Immune Imprints

Physiological and molecular alterations triggered by viral infections can have long-lasting functional consequences, allowing a more finely tuned response to subsequent pathogenic challenges.

An in vitro study using human bronchial epithelial cells infected multiple times with Human Rhinovirus, the most common viral agent responsible for the common cold, identified modification into DNA methylation pattern [94]. Authors found 77 genes and 122 CpGs islands “trainable” by multiple HRV infections. Collectively, these genes participate in transcriptional regulation and are linked to pathways involved in signal transduction, cell communication and differentiation, and extracellular matrix organization [94]. These alterations could lead to differential responses in further pathological exposures.

Severe RSV bronchiolitis has been linked to the onset of childhood wheezing and an increased risk of developing asthma later in life [95]. Severity of RSV disease is associated with DNA methylation signatures in saliva from infected infants [96]. In addition, patients who developed respiratory sequelae after RSV infection, such as recurrent wheezing, presented significant differentially methylated positions in genes associated with inflammatory response, compared to patients that completely recovered from infection [97]. Early-life RSV infection in mice induces long-term alterations in alveolar type 2 (AT2) cells, marked by epigenetic changes in the *Il33* gene promoter. These modifications likely drive persistent upregulation of *IL33* expression, promoting a pro-asthmatic environment that may contribute to increased lung disease severity later in life [98]. RSV infection in neonatal mice promotes altered immune responses through epigenetic and transcriptional programs in bone marrow-derived dendritic cells [99]. RSV infection induces the thymic stromal lymphopoietin expression that leads to upregulation of epigenetic enzymes such as histone demethylase that regulate transcriptional programs associated with the antiviral response mediated by type 1 interferon, implying an altered immune status when additional responses are initiated later in life. Interestingly, it has been demonstrated that bone marrow-derived dendritic cells are trained differently in male and female mice following early-life RSV [100]. ATAC-seq analysis revealed that females infected with RSV in early life exhibited increased chromatin accessibility near type I immune response genes compared to males, which might explain more male susceptibility to respiratory infections. Accordingly, RSV induces in mice the upregulation of Kdm5b, an H3K4 demethylase, and this is associated with dendritic cell type 1 interferon production, and it is key during secondary RSV infection [101]. RSV also modulates the expression of DNMT3A likely affecting DNA methylation profile [102]

The immune dysregulation observed in infants with respiratory diseases may result from epigenetic modifications, such as miRNAs, sncRNAs that regulate gene expression post-transcriptionally and represent potential disease biomarkers. For example, miR-146a-5p, a miRNA implicated in limiting excessive inflammation, is reduced in the airways of infants with severe lower respiratory bronchiolitis and with wheezing episodes [102].

The concept of developmental “windows” is useful for understanding how early-life exposures can influence immune and respiratory trajectories. During critical phases of development, environmental or infectious stimuli tend to produce stronger and more long-lasting effects on immune programming [103]. These windows may represent periods of vulnerability as well as opportunity, in which epigenetic remodeling is particularly active, allowing viral infections to modify inflammatory balance, airway responsiveness, and immune polarization [65,104]. Alterations occurring during these critical periods may persist beyond infancy and influence susceptibility to respiratory diseases over time [105]. Recognizing the existence of these windows helps contextualize the long-term consequences of early-life respiratory infections and supports the model illustrated in Figure 1 [67].

## 5. Future Directions

The neonatal period in life can serve as a “window of opportunity” to modulate epigenetic changes with the potential to prevent respiratory viral infections later in life. Among the various factors described in this review that can induce epigenetic modulation and shape immune responses toward more favorable outcomes during respiratory infections, the influence of the microbiota emerges as particularly significant for the development of future preventive and therapeutic interventions.

In this context, OM-85, a bacterial lysate derived from 21 respiratory tract bacterial strains—including *Haemophilus influenzae*, *Diplococcus pneumoniae*, *Klebsiella pneumoniae*, *Klebsiella ozaenae*, *Staphylococcus aureus*, *Streptococcus pyogenes*, *Streptococcus viridans*, and *Neisseria catarrhalis*—has gained prominence as an immunomodulatory intervention. OM-85, administered orally and considered safe, not only promotes terminal maturation of human dendritic cells with enhanced ability to activate T lymphocytes but also increases production of the Th1 cytokine IFN-γ while reducing IL-4 expression, associated with Th2 responses. These combined effects help strengthen natural defense mechanisms, protecting against respiratory infections, including RSV [106,107,108].

Another promising intervention is MV130, a mucosal polybacterial vaccine containing heat-inactivated Gram-positive (90%) and Gram-negative (10%) bacteria. Among these bacteria are *Streptococcus pneumoniae*, *Staphylococcus aureus*, *Staphylococcus epidermidis*, *Klebsiella pneumoniae*, *Moraxella catarrhalis*, and *Haemophilus influenzae* [109]. Clinical research indicates that patients with frequent respiratory infections exhibit a considerable reduction in the number of infectious episodes after using MV130, evidencing its potential as a preventive tool acting through immune system modulation.

Microbiota produced several metabolites that can influence the short-chain fatty acids, such as acetate, propionate and butyrate, which are the most studied examples and demonstrate to directly influence histone modification, the activity of regulatory enzymes, and transcription factors that play a role in immune responses [76]. Microbiota-derived acetate confers protection against respiratory syncytial virus (RSV) infection through activation of the GPR43 receptor and induction of a type I interferon–dependent antiviral response, contributing to reduced viral replication and pulmonary inflammation. In addition, acetate has been shown to induce RIG-I–mediated antiviral responses in cells from infants with RSV-associated bronchiolitis, reinforcing its role in early-life antiviral immunity. Complementarily, airway delivery of acetate enhances antiviral immunity during rhinovirus infection by increasing the production of type I and type III interferons and the expression of interferon-stimulated genes, resulting in lower viral loads in the early phases of infection and attenuation of late virus-induced inflammatory responses. In this way, microbial stimuli received since early life can shape more balanced inflammatory responses and increase resistance to viruses. This epigenetic reprogramming process emerges as a promising approach to reduce the severity and occurrence of respiratory infections [110,111,112].

Collectively, these findings reinforce that interventions aimed at microbiota modulation not only affect specific immune pathways but also have the ability to activate broader mechanisms of immune adaptation. This context aligns with the concept of trained immunity, in which microbial stimuli have the capacity to reprogram innate responses, strengthening defense against infections both associated and not associated with the initial trigger [54,55].

## 6. Conclusions

Early life is a highly vulnerable and dynamic period in which immune maturation is strongly influenced by environmental exposures, microbial signals, and infections. The major early-life factors, including prematurity, microbiota composition, and vaccination, shape antiviral responses through diverse epigenetic mechanisms—altering DNA methylation, chromatin accessibility, histone marks, and miRNA expression. Such modifications can either strengthen antiviral defenses or increase susceptibility to severe respiratory infection. Sex-specific differences in epigenetic reprogramming further underscore the complexity of these developmental processes [113]

Respiratory viruses themselves also leave lasting epigenetic and transcriptional imprints on airway and immune cells, influencing future responses and contributing to long-term outcomes such as recurrent wheeze and asthma. Together, these findings underscore that early-life epigenetic programming plays a central role in determining both acute disease severity and later respiratory health. Identifying stable epigenetic signatures and understanding how they can be modified may offer new opportunities for prevention and intervention in childhood respiratory disease.

Future studies should focus on identifying causal epigenetic signatures that predict susceptibility or protection, clarifying the persistence of epigenetic changes across childhood, and determining whether targeted interventions can beneficially reprogram immune trajectories.

## Data Availability

Data sharing is not applicable.

## Abbreviations

RSV	Respiratory Syncytial Virus
HRV	Human Rhinovirus
DNMT	DNA Methyltransferases
HATs	Histone Acetyltransferases
HDACs	Histone Deacetylases
NC	Non-coding
sncRNAs	Small Non-coding RNA
miRNAs	MicroRNA
ATAC-seq	Assay for Transposase-Accessible Chromatin
TET	Ten-Eleven Translocation
NK	Natural Killer
IFN	Interferon
IL	Interleukin
